# Chlamydiae in corals: shared functional potential despite broad taxonomic diversity

**DOI:** 10.1093/ismeco/ycae054

**Published:** 2024-04-15

**Authors:** Justin Maire, Astrid Collingro, Matthias Horn, Madeleine J H van Oppen

**Affiliations:** School of BioSciences, The University of Melbourne, Parkville 3010, VIC, Australia; Centre for Microbiology and Environmental Systems Science, University of Vienna, Vienna 1030, Austria; Centre for Microbiology and Environmental Systems Science, University of Vienna, Vienna 1030, Austria; School of BioSciences, The University of Melbourne, Parkville 3010, VIC, Australia; Australian Institute of Marine Science, PMB No 3, Townsville 4810, QLD, Australia

**Keywords:** coral, microbiome, chlamydiae, simkaniaceae, genomics, symbiosis, cnidarian, diversity

## Abstract

Cnidarians, such as corals and sea anemones, associate with a wide range of bacteria that have essential functions, including nutrient cycling and the production of antimicrobial compounds. Within cnidarians, bacteria can colonize all microhabitats including the tissues. Among them are obligate intracellular bacteria of the phylum Chlamydiota (chlamydiae) whose impact on cnidarian hosts and holobionts, especially corals, remain unknown. Here, we conducted a meta-analysis of previously published 16S rRNA gene metabarcoding data from cnidarians (e.g. coral, jellyfish, and anemones), eight metagenome-assembled genomes (MAGs) of coral-associated chlamydiae, and one MAG of jellyfish-associated chlamydiae to decipher their diversity and functional potential. While the metabarcoding dataset showed an enormous diversity of cnidarian-associated chlamydiae, six out of nine MAGs were affiliated with the Simkaniaceae family. The other three MAGs were assigned to the Parasimkaniaceae, Rhabdochlamydiaceae, and Anoxychlamydiaceae, respectively. All MAGs lacked the genes necessary for an independent existence, lacking any nucleotide or vitamin and most amino acid biosynthesis pathways. Hallmark chlamydial genes, such as a type III secretion system, nucleotide transporters, and genes for host interaction, were encoded in all MAGs. Together these observations suggest an obligate intracellular lifestyle of coral-associated chlamydiae. No unique genes were found in coral-associated chlamydiae, suggesting a lack of host specificity. Additional studies are needed to understand how chlamydiae interact with their coral host, and other microbes in coral holobionts. This first study of the diversity and functional potential of coral-associated chlamydiae improves our understanding of both the coral microbiome and the chlamydial lifestyle and host range.

## Introduction

The phylum Cnidaria comprises a wide range of early metazoans, including hard and soft corals, sea anemones, and jellyfish. These aquatic animals can have tremendous ecological value, such as scleractinian corals which form the foundation of coral reefs by building the reefs’ three-dimensional framework and driving its food web. Cnidarians associate with a wide range of microorganisms, which include protists, bacteria, archaea, viruses, and fungi [1–3]. Among them, bacteria have important functions, including protection against pathogens, and nitrogen and sulfur cycling [3–5]. Although the bacterial microbiome is highly diverse [6–8], most functional and genomic studies have focused on a few bacterial taxa, such as Proteobacteria, leaving most other taxa understudied. This includes the Chlamydiota (also known as chlamydiae), a phylum of obligate intracellular bacteria that infect a broad spectrum of eukaryotic hosts [9, 10]. All known chlamydiae have reduced genomes and a remarkably conserved biology; they are dependent on eukaryotic host cells, alternating between an infectious extracellular stage (referred to as elementary bodies) and an intracellular replicative stage (referred to as reticulate bodies) (see online supplementary material for a colour version of Fig. S1), and manipulate host cells through a type III secretion system (T3SS) [9]. While they are best known for being mammalian pathogens, chlamydiae, and especially members of the Simkaniaceae family, are regularly detected in metabarcoding data from scleractinian corals [11–14], octocorals [15, 16], jellyfish [17, 18], cultures of intracellular dinoflagellates (Symbiodiniaceae, found in the tissues of corals, sea anemones, and jellyfish) [19–21], as well as cultures of *Ostreobium* [22, 23], a green alga found in the skeleton of scleractinian corals.

Despite their prevalence, very little data exists on the diversity and function of cnidarian-associated chlamydiae, including whether they are pathogenic or mutualistic. This is likely due to their strictly host-associated lifestyle and generally low abundance in cnidarian holobionts [8]. So far, only one study has analyzed the genome and location of a chlamydial associate of corals: *Simkania* sp. Pac_F2b forms inclusions in the tentacles of the coral *Pocillopora acuta* and possesses all the chlamydial hallmark genes for host interaction (e.g. a type III secretion system [T3SS], nucleotide transporters, virulence effectors), although its role remains unclear [12]. More data is therefore needed to increase our understanding of the taxonomic and genomic diversity of cnidarian-associated chlamydiae and their impact on cnidarian health. Here, we conducted a meta-analysis of previously published cnidarian microbiome data and genomes of coral-associated chlamydiae. By analyzing their phylogeny and genomic content, we assessed whether chlamydiae detected in various hosts represent distinct chlamydial lineages, share functional potential, and which convergent traits have evolved in these chlamydiae.

## Materials and methods

### Analysis of cnidarian microbiome data

Five databases were obtained from a previously published meta-analysis of 186 cnidarian and cultured Symbiodiniaceae microbiome studies [7]. Combined, the five databases contain 12 307 samples, including 10 219 Hexacorallia, 1434 Octocorallia, 356 jellyfish (Cubozoa, Scyphozoa), 53 Hydrozoa, and 245 Symbiodiniaceae. Detailed metadata is available in the original study [7]. The five datasets each regroup metabarcoding data based on the hypervariable region of the 16S rRNA gene that was targeted (V1-V2, V3, V4, V5-V6, and V7-V8, respectively). The five datasets were analyzed independently. The datasets were analyzed in RStudio v2023.06.2 using the phyloseq package [24]. Amplicon sequence variants (ASVs) affiliating with the phylum Chlamydiota were identified in each dataset. Their relative abundance and prevalence (calculated as the percentage of samples within a family that harbors at least one chlamydial ASV – very low prevalences are to be taken with caution, as the presence of very few reads could be the result of contaminations or sequencing/processing errors) within cnidarian families and symbiodiniacean genera, as well as according to cnidarian health status was plotted in GraphPad Prism. Cnidarian health status reflected what was reported in each study, and was already included in the published dataset metadata [7].

Chlamydial ASVs from each dataset were aligned to a reference alignment with SINA v1.7.2 [25] and subsequently clustered into genus-level OTUs with usearch v11.0.667 (−cluster_smallmem –id 0.95 -query_cov 0.9) [26]. The chlamydial 16S rRNA gene reference tree was built based on comprehensive near full-length (>1200 nt length) 16S rRNA gene data from SILVA v138 SSU Ref NR 99 and sequences from Schulz *et al*. 2017 [27]. Sequences were dereplicated at 99% sequence identity. The sequence alignment was performed with SINA because it takes into account the secondary structure of ribosomal RNAs. The use of the SILVA database and SINA aligner are standard in 16S rRNA gene based phylogenetic studies. The resulting datasets had the following OTUs: V1-V2 region: n = 4 (7 before clustering), V3: n = 363 (700 before clustering), V4: n = 939 (2638 before clustering), V5-V6: n = 367 (727 before clustering), and V7-V8: n = 68 (96 before clustering). Centroid sequences of each cluster were then added to a chlamydial 16S rRNA gene reference multiple sequence alignment with MAFFT v7.5.20 (—addfragments –keeplength). The aligned centroid sequences for each dataset were separately added to a chlamydial 16S rRNA gene phylogenetic tree with EPA-ng v0.3.8 [28] using the SYM + I + R10 model. The full-length 16S rRNA gene reference tree was based on a previously published phylogeny [29] and updated with 25 full-length 16S rRNA gene sequences encoded in chlamydial MAGs published since. The reference tree was inferred with IQ-TREE v2.2.5 [30] with 1000 ultrafast bootstrap replicates [31] and 1000 replicates of the SH-like approximate likelihood ratio test [32] under the SYM + I + R10 model. The resulting phylogenetic trees with added centroid sequences from the cnidarian datasets were rooted using Planctomycetota and Verrucomicrobiota sequences as outgroup and visualized with iTOL v6.8.1 [33].

### Phylogenetic analyses of metagenome-assembled genomes of cnidarian-associated chlamydiae

Nine metagenome-assembled genomes (MAGs) of cnidarian-associated chlamydiae were recovered from the GenBank/ENA/DDBJ database through the NCBI database query interface. Eight were associated with corals, while one was associated with jellyfish. The levels of completeness and contamination were assessed using CheckM2 v1.0.2 [34]. Taxonomic assignment was carried out with GTDB-Tk v2.1.0 [35] using the “classify_wf” workflow.

For comparative and phylogenetic analyses, a reference dataset of high quality chlamydial genomes (of any host) based on Dharamshi *et al*. (2022) [36] was used, and updated by adding newly published genomes belonging to the chlamydiae available on GenBank/ENA/DDBJ on 29.05.2023 (Table S1). Only genomes with a completeness >70%, contamination <5% (both determined with CheckM2 v1.0.2 [34]), and average nucleotide identities <95% (determined with FastANI v1.33 [34]) were considered for the final reference dataset. Five MAGs with lower completeness were included: Waddliaceae bacterium CSSed165cm_440, to increase the Waddliaceae diversity (only in the OG analysis); Chlamydiia bacterium NC_groundwater_921_Pr1_S-0.2um, to increase the CC-III diversity (only in the OG analysis); Chlamydiota bacterium SZUA-184, to increase the Anoxychlamydiaceae diversity (only in the OG analysis); Chlamydiae bacterium 3300011418-10, to increase the Sororchlamydiaceae diversity; and uncultured bacterium EVH04_Bin2, to increase the diversity of cnidarian-associated MAGs. The final reference dataset included 169 chlamydial genomes (of any host) and 89 genomes from Planctomycetota and Verrucomicrobiota serving as outgroup (Table S1).

To obtain the phylogenetic affiliation of the cnidarian-associated chlamydial MAGs, a set of 15 conserved non-supervised orthologous groups (NOGs) was used (Table S2). These 15 NOGs are known to retrieve the same topology for chlamydial phylogeny as the application of larger protein sets [36]. Proteins of the chlamydial and Planctomycetota-Verrucomicrobiota outgroup genomes belonging to the 15 NOGs were aligned with MAFFT v7.520 L-INS-i [37]. The resulting single protein alignments were subsequently trimmed with BMGE v2.0 [38] and concatenated. Four chlamydial MAGs with <50% of the NOGs present were excluded from phylogenetic analysis (Table S1). Maximum likelihood phylogeny was inferred with IQ-TREE v2.2.5 [30] with 1000 ultrafast bootstrap replicates [31] and 1000 replicates of the SH-like approximate likelihood ratio test [32] under the LG + F + I + R10 model. The resulting phylogenetic tree was rooted using the outgroup and visualized with iTOL v6.8.1 [33].

### Genome annotation and metabolic pathway reconstruction

Genome annotation was performed with Bakta v1.7.0 [39] and eggNOG-mapper v2.1.11 [40]. Metabolic pathways, transport systems, and secretion systems were annotated via METABOLIC-G v4.0 [41] as previously described [42], applying “-m-cutoff 0.5” to include pathways which are ≥50% complete. The completeness of metabolic pathways (Kyoto Encyclopedia of Genes and Genomes [KEGG] module database, https://www.genome.jp/kegg/module.html), transporters and secretion systems was estimated in EnrichM v0.6.4 [43]. KEGG modules of interest were then plotted in GraphPad Prism and only modules which were ≥ 75% complete were considered functional. The presence of specific genes of interest (virulence genes, T3SS) were investigated using BLAST and known sequences from the complete, high-quality genomes of either *Simkania negevensis* (unknown host) [44] or *Chlamydia trachomatis* (mammalian pathogen) [45] (Table S3). Secondary metabolites were predicted using antiSMASH v7.0.0 [46].

### Analysis of orthologous groups of proteins

For further comparative analysis of the chlamydial genomes (see Table S1), all encoded protein sequences were clustered into orthologous groups (OGs) with OrthoFinder v2.5.5 [47] under default parameters. OGs and their respective eggNOG annotations were merged in RStudio v2023.06.2 and analyzed. Protein sequences of six OGs were exclusively present in the genus *Simkania*, but did not show any sequence similarity to known proteins in public databases. Thus, the structures of representative sequences of these OGs were predicted with AlphaFold v2.3.2 [48] and visualized with UCSF ChimeraX v1.6.1 [49]. The resulting best-ranked protein structure models were then searched with structure search against RCSB protein data bank [50]. Only hits with global pLDDT >70 were considered in the results. OG classifications were also used to detect amino acid transporters and peptidases.

## Results

### Occurrence and prevalence of chlamydiae in cnidarians

**Figure 1:**
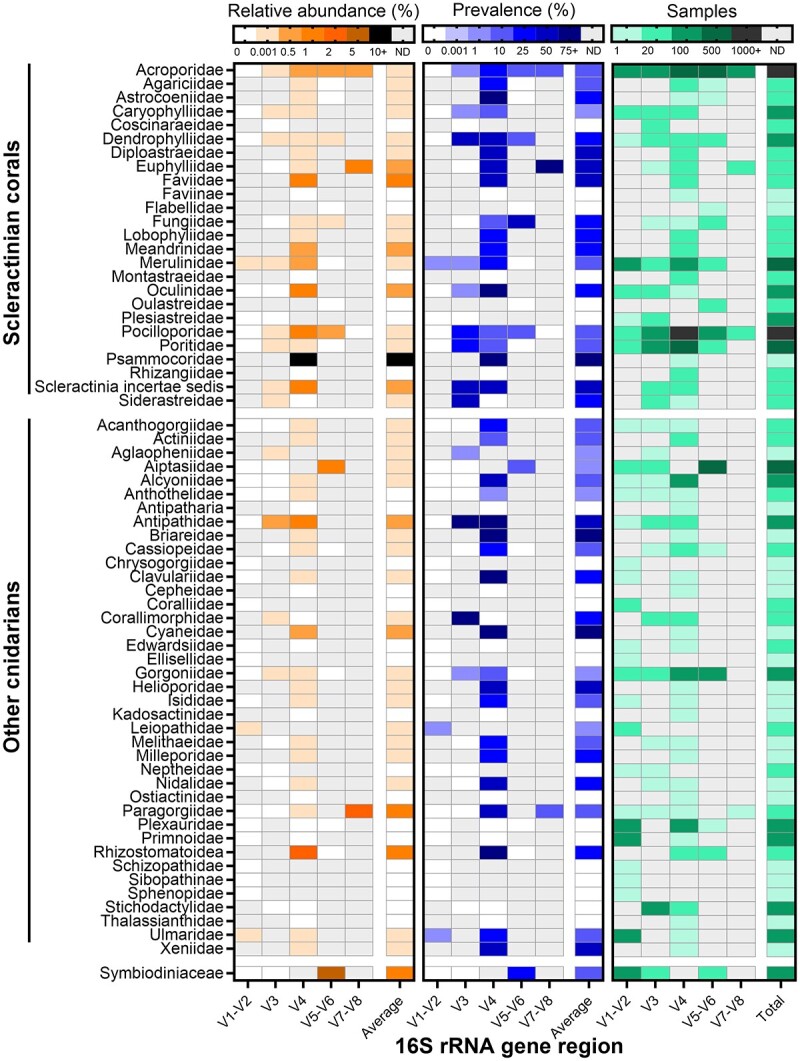
Relative abundance (left) and prevalence (middle) of chlamydiae amplicon sequence variants (ASVs) across cnidarian families and cultured Symbiodiniaceae. Prevalence was calculated as the number of samples per family that harbors at least one chlamydial ASV. The number of individual samples for each cell is also provided (right). Data was obtained from a meta-analysis of 186 16S rRNA gene metabarcoding studies [7]. ND: no data.

By analyzing datasets from 186 cnidarian and Symbiodiniaceae microbiome studies [7], we found that reads assigned to the phylum Chlamydiota are widespread in these host taxa, being detected in 1925 out of >12 000 samples, although usually at a low relative abundance (< 2% for all families except Psammocoridae, Fig. 1). The highest relative abundance was in the scleractinian family, Psammocoridae (average 32% chlamydiae), although this data came from only one study (seven samples across two timepoints) and showed high variation between the two timepoints (43–87% for the first timepoint, 0.4–1.6% for the second timepoint) [51]. The prevalence of chlamydiae was variable, though consistently high in certain scleractinian families, i.e., the Acroporidae (29% prevalence across 742 samples in the V4 region), Pocilloporidae (22% prevalence across 1128 samples in the V4 region), and Dendrophylliidae (72–75% prevalence across 136 samples in the V3, V4, and V5-V6 regions); high prevalence was also observed in one actiniarian family, Aiptasiidae (24% prevalence across 501 samples in the V5-V6 region). In addition, chlamydiae were found in both healthy (0.14–2.39% across all cnidarians) and unhealthy (i.e., stressed, bleached, damaged, or diseased, as reported in the original studies; 0.01–1.97%) samples, often being slightly more abundant in healthy cnidarians (see online supplementary material for a colour version of Fig. S2). This shows that, while widespread, chlamydiae are not specifically associated with unhealthy or diseased cnidarians.

### Diversity of cnidarian- and symbiodiniaceae-associated chlamydiae

**Figure 2:**
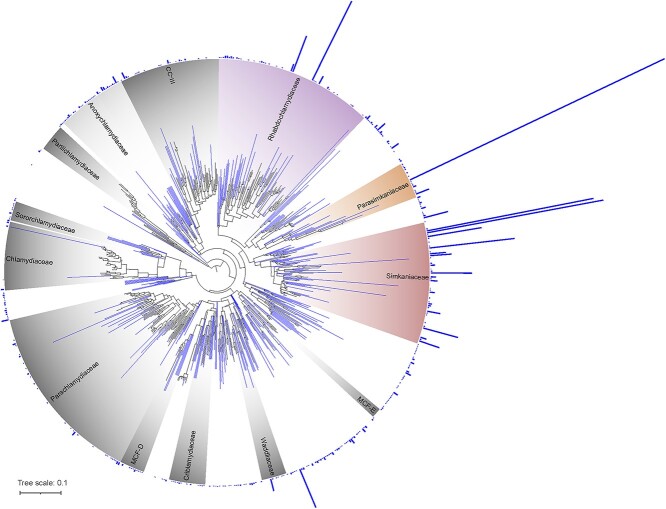
Diversity of cnidarian- and Symbiodiniaceae-associated chlamydiae. Maximum likelihood phylogeny of the 16S rRNA gene (V3 region) of 363 chlamydial genus-level OTUs. Blue branches represent chlamydial OTUs found in a dataset of 43 cnidarian and Symbiodiniaceae 16S rRNA gene (V3 region) metabarcoding studies [7], while bars outside the circle represent the total read number (2–10 640 reads) of each OTU. OTUs with no outside bars are reference sequences. Trees for four other 16S rRNA gene regions are available, see online supplementary material for a colour version of Figs S3–S6. Colored taxa (Simkaniaceae, Parasimkaniaceae, Rhabdochlamydiaceae, and Anoxychlamydiaceae) are families from which we obtained cnidarian-associated MAGs. MCF: metagenomic chlamydial family; CC-III: chlamydiae clade III.

To gain insights into the overall diversity of cnidarian- and Symbiodiniaceae-associated chlamydiae, we extracted the chlamydial ASVs from the metabarcoding datasets [7] and clustered the ASVs present in each dataset at genus-level identity (95%). The resulting cluster centroid sequences for each variable region of the 16S rRNA gene dataset were added to a chlamydial 16S rRNA gene reference tree. An enormous diversity of chlamydiae was detected in cnidarian and Symbiodiniaceae microbiomes (Figs 2 and S3–S6), with 1741 genus-level chlamydial OTUs obtained from the five datasets. The families Simkaniaceae, Parasimkaniaceae, and Rhabdochlamydiaceae contained the most abundant ASVs, though this may be impacted by the different sequencing depths of each study, as well as known biases from the most commonly used universal primers against chlamydiae (e.g. 515F/806R of the V4 region, or 1391R of the V8 region [52]). Only two chlamydial families were not found in cnidarians: Parilichlamydiaceae and Piscichlamydiaceae, while only six Chlamydiaceae ASVs were detected; these are all known to specifically infect chordate hosts.

### Metagenome-assembled genomes of cnidarian-associated chlamydiae

**Figure 3:**
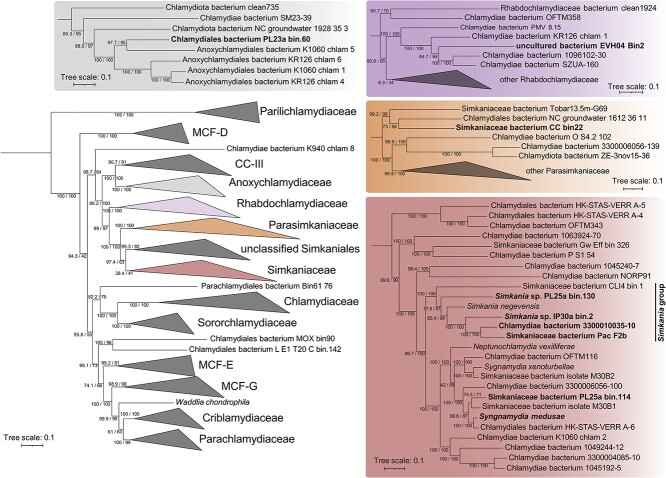
Chlamydial maximum likelihood phylogeny based on 15 conserved NOGs in 165 chlamydial genomes**.** Cnidarian-associated chlamydiae are in bold. Confidence values based on 1000 ultrafast bootstrap replicates and 1000 replicates of the SH-like approximate likelihood ratio test are provided. Additional data on the reference genomes is available in Table S1. MCF: Metagenomic chlamydial family; CC-III: Chlamydiae clade III.

To better understand the nature of cnidarian-chlamydiae interactions, we analyzed nine MAGs of cnidarian-associated chlamydiae detected previously in metagenomic studies (Tables 1 and S4). Seven MAGs were obtained from scleractinian corals, one from an octocoral (Gorgoniidae, *Eunicella verrucosa*), and one from a jellyfish. Because eight out of nine MAGs were obtained from corals, we will restrict our conclusions to corals, rather than include the whole Cnidarian phylum. MAG size ranged from 0.88 to 2.14 Mb with estimated complete genome sizes ranging between 1.3 and 2.2 Mb. One MAG (EVH04_Bin2) was only 53.33% complete – it was still included in our analyses as it was the MAG obtained from a non-scleractinian coral, though we were careful with our interpretations. All other MAGs ranged from 78.84% to 100% in completeness. Contamination ranged from 0.07% to 3.14%. All but two (Pac_F2b [12] and *S. medusae* [18]) were not analyzed in their original publication. To assess their taxonomy, we created a phylogenetic tree based on 15 conserved marker genes (Table S2), along with 161 other chlamydial genomes (Fig. 3, Table S1). Six MAGs affiliated with members of the Simkaniaceae family, including four in the *Simkania* genus, according to GTDB-Tk classification, along with the type species *Simkania negevensis* and a MAG obtained from a Great Barrier Reef sponge (CLI4_bin_1 [53]). The other three MAGs belonged, respectively, to Rhabdochlamydiacaeae, Anoxychlamydiaceae, and the recently described Parasimkaniaceae, closely related to Simkaniaceae.

**Table 1:** Characteristics of nine published cnidarian-associated chlamydial MAGs. Additional details are available in Table S4.

**Genome ID**	**Host**	**Tissue**	**Location**	**Size (Mb)**	**Family**	**Study**
3300010035–10	*Cyphastrea* sp.	Unknown	Lord Howe Island, Australia	1.32	Simkaniaceae	[54]
CC_bin22	*Acropora digitifera*	Surrounding seawater	Daya Bay, Shenzhen, China	1.80	Parasimkaniaceae	[55]
EVH04_Bin2	*Eunicella verrucosa*	Unknown	Algarve, Pedra da Greta, Portugal	0.88	Rhabdochlamydiaceae	[56]
IP30a_bin.2	*Isopora pallifera*	Skeleton	Heron Island, Australia	1.06	Simkaniaceae	[57]
Pac_F2b	*Pocillopora acuta*	Tentacle epidermis	Feather Reef, Australia	1.25	Simkaniaceae	[12]
PL23a_bin.60	*Porites lutea*	Skeleton	Heron Island, Australia	1.79	Anoxychlamydiaceae	[57]
PL25a_bin.114	*P. lutea*	Skeleton	Heron Island, Australia	1.88	Simkaniaceae	[57]
PL25a_bin.130	*P. lutea*	Skeleton	Heron Island, Australia	1.59	Simkaniaceae	[57]
SYNMED	*Cotylorhoiza tuberculata*	Gastric cavity	Alcúdia Bay, Spain	2.14	Simkaniaceae	[18]

Additional details are available in Table S4.

### Functional potential of coral-associated chlamydiae

**Figure 4:**
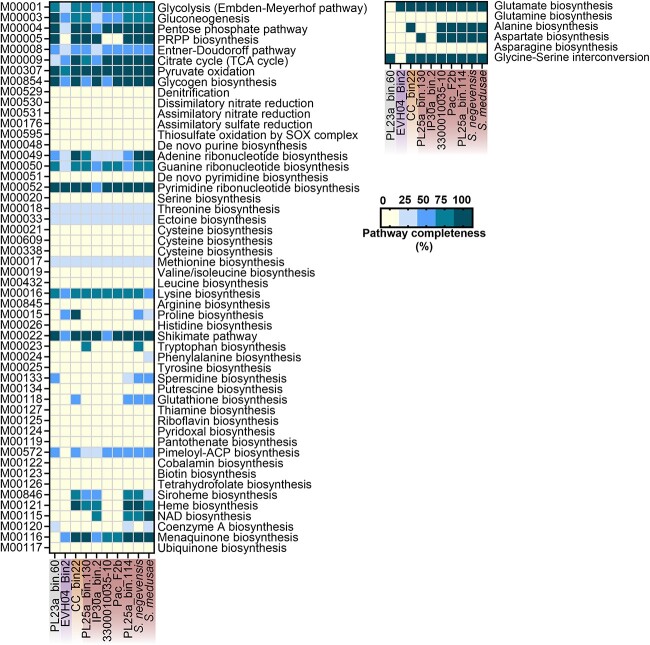
Metabolic potential encoded in eight coral-associated and one jellyfish-associated chlamydial MAGs and *S. negevensis*. Genome names are colored according to the chlamydial family they belong to in Fig. 3; grey: Anoxychlamydiaceae; purple: Rhabdochlamydiaceae; orange: Parasimkaniaceae; red: Simkaniaceae. The completeness of metabolic pathways (left panel; KEGG module database) of interest were annotated using METABOLIC-G and pathway completeness (in %) was estimated in EnrichM. The presence of genes involved in additional amino acid synthesis (right panel) was checked manually through the KEGG reconstruct tool. Detailed completeness is available in Table S5.

Metabolic potential was assessed based on KEGG annotations and pathways (Fig. 4, Table S5). The complete genome of *S. negevensis* [44] was used as a reference. Pathways that were ≥ 75% complete were assumed to be functional, although we acknowledge that (i) these analyses are only predictions and need to be confirmed experimentally and (ii) a single missing gene may render an entire pathway non-functional. All MAGs showed reduced metabolic abilities, lacking pathways for the biosynthesis of nucleotides, vitamins, and most amino acids (only genes for glutamate, aspartate, lysine, and alanine biosynthesis were found, as well as glycine-serine interconversion, and the shikimate pathway). Further, all MAGs possessed oligopeptide ABC transporters, specific amino acid transporters (e.g. for tyrosine and proline), as well as peptidases (Table S6), which may allow them to degrade and import oligopeptides and amino acids. Most MAGs possessed the genes necessary for glycolysis, the tricarboxylic acid cycle, and the pentose phosphate pathway (see detailed completeness data in Table S5). The pathways were missing in EVH04_Bin2 (Rhabdochlamydiaceae) and IP30a_bin.2 (Simkaniaceae), which may be explained by their level of incompleteness (53.33% and 80.41% complete, respectively). The TCA was also missing from PL23a_bin.60 (Anoxychlamydiaceae), which instead possessed genes typically involved in anaerobiosis, such as genes encoding pyruvate:ferredoxin (flavodoxin) oxidoreductase (UniRef: Q2RMD6), flavodoxin (UniRef: A0A1F9ABW2), ferredoxin (UniRef: A0A6M2A157), and the hydrogenase HydA (UniRef: A0A6M1YVE0 and A0A6M2AB78).

The pathways for the biosynthesis of two co-factors were complete in some MAGs: menaquinone (in six MAGs and *S. negevensis*, all part of the Simkaniaceae family) and heme (in five MAGs and *S. negevensis*, all part of the Simkaniaceae family). Yet, all 10 genes of the heme biosynthesis pathway were missing from two other Simkaniaceae MAGs (3300010035–10 and Pac_F2b). Because of the large number of genes missing, this is unlikely a consequence of MAG incompleteness. Finally, we wanted to infer the potential production of secondary metabolites and queried their presence in antiSMASH (Table S7). Four MAGs did not contain any predicted secondary metabolite gene cluster, while four (PL25a_bin.114, PL23a_bin.60, 3300010035-10, CC_bin22) possessed one each, and *S. medusae* possessed three, all with putative antimicrobial properties. By contrast, the genome of *S. negevensis* was predicted to synthesize three secondary metabolites.

**Figure 5:**
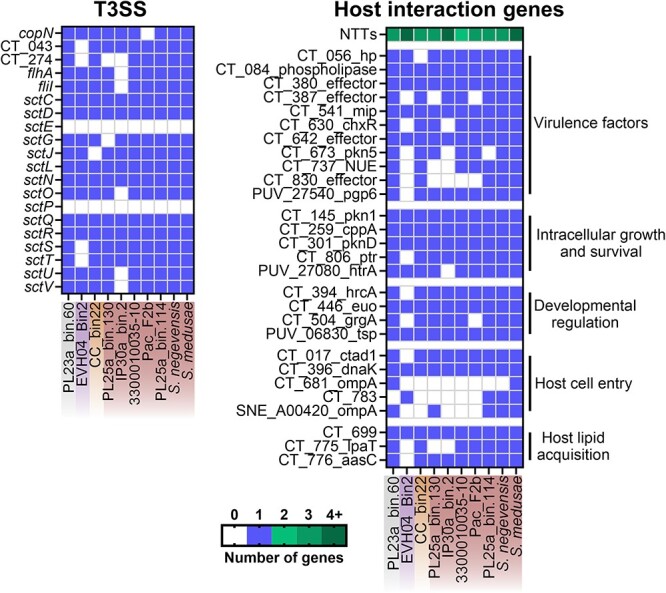
Important genes for host interactions encoded in eight coral-associated and one jellyfish-associated chlamydial genomes and *S. negevensis*. Genome names are colored according to the chlamydial family they belong to in Fig. 3; grey: Anoxychlamydiaceae; purple: Rhabdochlamydiaceae; orange: Parasimkaniaceae; red: Simkaniaceae. Genes important for host interactions from the complete genomes of *C. Trachomatis, Parachlamydia acanthamoebae,* and *S. Negevensis* were blasted against the nine coral-associated chlamydial MAGs to assess their presence. Additional information on the selected genes is available in Table S3.

The pathogenic potential of coral-associated chlamydiae was examined by similarity searches against hallmark chlamydial virulence-associated genes (Fig. 5, Table S3) [29]. All MAGs had a complete T3SS, except for EVH04_Bin2 and IP30a_bin.2. The T3SS is a key molecular machinery that allows for translocation of effectors into eukaryotic host cells. However, none had genes to build a flagellar apparatus, which is at odds with other marine chlamydiae [58]. All MAGs encoded most chlamydial lifestyle- and virulence-associated genes that are present in other non-chordate infecting chlamydiae. This includes nucleotide transporters (ATP and nucleotide import), adhesins, T3SS effectors, Ser/Thr kinases (host cell modulation), and developmental regulators such as *euo*, which governs the transition between the two chlamydial developmental stages (see online supplementary material for a colour version of Fig. S1). Two genes involved in host cell lipid acquisition (*lpaT, aasC* [59]) were missing from one and three MAGs, respectively (EVH04_Bin2, IP30a_bin.2, and PL25a_bin.130), suggesting these MAGs may use a different mechanism for lipid acquisition. Finally, four potential T3SS effectors (*NUE,* CT_387, CT_783, and CT_830) were missing from 3 to 6 MAGs, with PL25a_bin.130 missing all four.

### Genes unique to marine invertebrate-associated *Simkania*

To explore the presence of taxonomically restricted genes, we first queried the presence of genes specific to the nine cnidarian-associated MAGs that are not found in other chlamydiae. Genes common to all nine chlamydial genomes were also present in at least one of the other chlamydial genomes in the dataset (Table S1), showing a lack of genomic specificity in coral-associated chlamydiae compared to other chlamydiae. This may be due to the taxonomic breadth represented by the cnidarian coral-associated chlamydiae (i.e. three distantly related families). Therefore, we also analyzed the marine *Simkania* group, including one sponge-associated and four coral-associated *Simkania* (Fig. 3). We found six genes that were present in at least four of the five MAGs and that were absent from all other chlamydiae genomes (Table 2). Because all six genes were predicted to encode hypothetical proteins without any known motifs, we predicted their structure using AlphaFold and queried the RCSB Protein Data Bank to identify structurally similar proteins (Table 2 and see online supplementary material for a colour version of Fig. S7). Two out of six genes resulted in reliable structural predictions (pLDDT >70) and had hits in the RCSB Protein Data Bank which also had structural predictions with a high confidence (pLDDT >70). These included a peroxisomal biogenesis factor (involved in peroxisome formation) and an outer membrane protein.

**Table 2:** List of genes present in at least four out of the five MAGs of the marine *Simkania* group and absent from all other chlamydiae.

Orthogroup	Protein product^a^	Structure pLDDT^b^ (global)	Closest proteins^c^
OG0004258	WP_013943428.1	*43.2*	-
OG0004395	WP_013944476.1	76.1	Uncharacterized protein AF_AFU7PWL2F1
			peroxisomal biogenesis factor 11C AF_AFQ96HA9F1
OG0004813	WP_013944279.1	74.8	Uncharacterized protein AF_AFU7PW52F1
			YnfB AF_AFB5RAG2F1
OG0004814	WP_013943429.1	*44.9*	–
OG0004815	WP_013943431.1	*60.8*	–
OG0004816	WP_013944307.1	*43.2*	–

The closest proteins based on structural predictions are provided.

a Product in the reference genome of *Simkania negevensis* (NC_015713.1). All six orthogroups were initially annotated as hypothetical proteins.

b pLDDT: predicted local distance difference test score (high confidence if pLDDT >70). Structures with low confidence are italicized.

c Only hits with pLDDT >70 were considered.

## Discussion

Chlamydiae are among the most successful microbial symbionts of eukaryotes and infect a wide array of hosts, from protists to mammals [10]. This includes marine hosts and environments, with vast amounts of chlamydial sequences and genomes being retrieved from marine animals and sediments (chlamydiae may temporarily be extracellular as elementary bodies) [29, 36]. Despite being abundant and prevalent in cnidarians, cnidarian-associated chlamydiae have remained largely understudied. Here, we analyzed previously published data from cnidarian and Symbiodiniaceae microbiome studies and nine chlamydial MAGs sequenced from cnidarian hosts (corals and jellyfish), to increase our understanding of both chlamydial and cnidarian biology, and lay the foundations for further studies on cnidarian–chlamydiae interactions. The sequences derived from more than 12 000 cnidarian and Symbiodiniaceae samples [7] enabled an overview of the presence, prevalence, and diversity of chlamydiae in cnidarian holobionts. Cnidarian-associated chlamydiae affiliated with most described chlamydial families, with the exception of members of the chordate-infecting Piscichlamydiaceae, Parilichlamydiaceae, and Chlamydiaceae. High chlamydial diversity has also been described for sponges [36], pointing towards potentially deep evolutionary relationships with these two groups of early metazoans.

Seven out of nine MAGs were members of the Simkaniaceae and Parasimkaniaceae families, which are often found to be highly abundant in cnidarian metabarcoding studies [7, 11, 14]. The Simkaniaceae family contains many marine representatives derived from jellyfish, sponges, fish, and marine sediments [29, 36], with a recent meta-analysis showing that ~60% of published Simkaniaceae 16S rRNA sequences originated from marine environments, with another ~30% from animal hosts (host environment unspecified) and engineered environments (e.g. bio-reactors, wastewater) [29]. This shows that marine ecosystems harbor many suitable hosts for Simkaniaceae. Strikingly, four coral-associated MAGs, along with a sponge-associated MAG, are close relatives of the type species *S. negevensis* and likely belong to the *Simkania* genus. *S. negevensis* was initially discovered as a laboratory contaminant of human cell cultures in Israel [60], although its original host was never elucidated. Its phylogenetic relatedness with coral- and sponge-associated Simkaniaceae highlighted in our study points to a marine origin of *S. negevensis*. *S. negevensis* is one of the few chlamydiae successfully cultured in a lab setting (inside a host), and its ability to replicate in a marine environment or host should be checked.

Two additional coral-associated MAGs belonged to the Rhabdochlamydiaceae and Anoxychlamydiaceae. The Anoxychlamydiaceae MAG (PL23a_bin.60) was obtained from coral skeleton, a few centimeters underneath the algal (*Ostreobium)* layer [57]. The skeleton is anoxic past the first few centimeters [61], and probably up to the coral tissue layer at night, when phototrophic endoliths (e.g. *Ostreobium*, Cyanobacteria) do not photosynthesize [62], and could therefore support anaerobic microorganisms such as Anoxychlamydiaceae [63]. This is consistent with the incompleteness of the TCA cycle, an aerobic process, in PL23a_bin.60 and the presence of genes encoding pyruvate:ferredoxin (flavodoxin) oxidoreductase, flavodoxin and rubredoxin observed also in other Anoxychlamydiaceae and are consistent with anaerobic metabolism [63].

Comparative gene content analysis did not detect any genes that are restricted to coral-associated chlamydiae. A similar conclusion was drawn from a recent analysis of sponge-associated chlamydiae, which found that gene content was related to phylogenetic affiliation rather than to an ecological association with their host [36]. Broader metabolic pathway reconstruction corroborated this lack of specificity, as most coral-associated chlamydiae shared metabolic and host interaction abilities with chlamydiae from other hosts. Within the marine *Simkania* group, however, the comparative analysis identified six genes that are not found in any other chlamydiae. Based on structural predictions with AlphaFold, functions could be hypothesized for two of the six genes. OG0004395 may represent peroxisomal biogenesis factor-like proteins, which could interfere with host peroxisome size and fission. Eukaryotic peroxisomes play a role in clearing bacterial infections by assisting with phagocytosis [64]. For instance, *C. trachomatis* interacts with host peroxisomes, possibly for phospholipid synthesis [65]. Marine *Simkania* may therefore interact with host peroxisomes for cell growth or phagocytosis escape. OG0004813 includes proteins most closely related to bacterial outer membrane proteins, and thus these proteins may be involved in host cell adhesion. A key difference among the marine *Simkania* was that the heme biosynthesis pathway was completely absent from two out of four *Simkania* MAGs. This stark difference in closely related organisms may be explained by factors unrelated to chlamydial or host taxonomy, such as the host environment (e.g. depth, light conditions, and temperature variability) and which host microhabitat the *Simkania* reside in (e.g. gastrodermis, epidermis, and skeleton). Mutations in the *hemG* gene, involved in heme synthesis, were associated with increased infectivity in *C. trachomatis* [66], suggesting that the presence or absence of the heme biosynthesis pathway may lead to different infection outcomes. Despite this difference, the overall gene content similarities among marine *Simkania* are consistent with previous studies finding that chlamydiae in general have a large core genome [29, 44, 67, 68], with similar metabolic abilities and lifestyle features, such as energy parasitism and a biphasic developmental cycle. The lack of recognizable host-driven genomic convergence in several chlamydial groups may provide more host flexibility and explain the wide range of eukaryotic hosts they have successfully formed associations with.

While genomic analyses confirmed that coral-associated chlamydiae possess all the genes needed for the chlamydial intracellular, parasitic lifestyle, it remains unclear whether they are beneficial or detrimental to their hosts, as it is in many other organisms, and if their presence is an indicator of holobiont health. We found that chlamydiae prevalence was similar between healthy and unhealthy corals, and no coral disease is known to be caused by chlamydiae. Bleached corals did not show increased chlamydial abundance either, suggesting chlamydiae may not opportunistically infect stressed animals, as seen for example with the coral pathogen *Vibrio coralliilyticus* [69]. Thus, as for sponges [36], negative impacts of chlamydiae infections in corals have not been observed. Potentially beneficial functions, however, remain elusive, with the exception of the production of secondary metabolites (found in only five out of nine MAGs) which may be involved in chemical defense against other microorganisms. They could therefore function as defensive symbionts, as recently shown in protist hosts where chlamydiae provide protection against *Legionella* [70, 71] or giant viruses [72]. This would however require every cell of exposed tissue to harbor chlamydiae. Functional studies should be conducted, where holobiont fitness metrics must be compared between infected and uninfected colonies of the same species and geographic location (e.g. *Acropora loripes* from Davies Reef, Australia [11]). In combination with gene expression analyses of both host and chlamydiae, through metatranscriptomics or spatial transcriptomics, such experiments may shed light on coral–chlamydiae interactions.

Finally, while chlamydiae are abundantly found in cnidarian metabarcoding studies, cnidarian samples often contain symbiotic microeukaryotes, such as fungi and protists (e.g. Symbiodiniaceae, *Ostreobium*, corallicolids, and chromerids) [2, 73]. Chlamydiae have been detected in laboratory cultures of both Symbiodiniaceae and *Ostreobium* [19–23]. Whether chlamydiae can infect these protists *in hospite* remains unknown, although *S. medusae* was visualized inside ciliate cells in jellyfish samples [18]. Four MAGs analyzed in our study were isolated from the coral skeleton [57], and may therefore be derived from *Ostreobium* or other endolithic protists. One MAG (Pac_F2b) was obtained from bacterial clusters excised from coral epidermis and the strain represented by this MAG therefore likely infects coral cells. Protist-associated chlamydiae (e.g. members of the Amoebachlamydiales) have larger genomes (~2–3 Mb) compared to animal-associated chlamydiae (~1–2 Mb) [44, 67]. Larger genomes may offer more flexibility to infect unicellular organisms, which provide less stable conditions than multicellular organisms. Except for *S. medusae*, which may infect ciliates inside jellyfish [18], the MAGs analyzed in our study were all <2 Mb, suggesting they are coral- rather than protist-associated. Further studies combining genomic and spatial analyses are needed to fully elucidate the hosts and functions of chlamydiae within cnidarian holobionts [8, 12, 13, 74, 75].

In conclusion, we provide the first genomic meta-analysis of coral-, and to a lesser extent cnidarian-associated chlamydiae. Coral-associated chlamydiae are highly similar to other chlamydiae and we found little genomic specialization. Broader sequencing of coral and cnidarian microbiomes (genomic, transcriptomic, and proteomic sequencing), as well as experimental and functional studies, will be needed to fully understand the breadth of cnidarian–chlamydiae interactions, including where they sit on the mutualism–parasitism continuum and whether they are an indicator of cnidarian health. Enrichment methods should be employed to maximize bacterial reads in cnidarian metagenomic sequencing data, which often contain disproportionate amounts of Symbiodiniaceae reads, leaving little sequencing depth for bacterial associates [8, 76, 77]. A wider range of hosts needs to be targeted, including jellyfish and sea anemones, as our study relied mostly on coral data, therefore limiting our conclusions. Seawater and sediment samples also need to be investigated in order to assess the host specificity of cnidarian-associated chlamydiae. Our research adds to the immense diversity of chlamydial hosts already described and improves our understanding of chlamydial evolution, and the broader evolution of intracellular symbioses.

## Supplementary Material

supplementary_information_revi_ycae054

Table_S1-genome_dataset_ycae054

Table_S3-virulence_gene_annotation-clean_ycae054

Table_S4-MAG_info-revision_ycae054

Table_S5-pathwaycomp_ycae054

Table_S6-AA-acquisition_ycae054

Table_S7-antismash_ycae054

## Data Availability

All data used in this study was previously published (See Tables S1 and S4 for accession numbers).
